# Knockout of *OsGAPDHC7* Gene Encoding Cytosolic Glyceraldehyde-3-Phosphate Dehydrogenase Affects Energy Metabolism in Rice Seeds

**DOI:** 10.3390/ijms252212470

**Published:** 2024-11-20

**Authors:** Jin-Young Kim, Ye-Ji Lee, Hyo-Ju Lee, Ji-Yun Go, Hye-Mi Lee, Jin-Shil Park, Yong-Gu Cho, Yu-Jin Jung, Kwon-Kyoo Kang

**Affiliations:** 1 Division of Horticultural Biotechnology, Hankyong National University, Anseong 17579, Republic of Korea; zino@hknu.ac.kr (J.-Y.K.); lyj7776@naver.com (Y.-J.L.); ju950114@naver.com (H.-J.L.); abc_772@naver.com (H.-M.L.); qkrwlstlf0822@naver.com (J.-S.P.); yuyu1216@hknu.ac.kr (Y.-J.J.); 2 Department of Bio-Environmental Chemistry, College of Agriculture and Life Sciences, Chungnam National University, Daejeon 34134, Republic of Korea; jy.go2369@gmail.com; 3 Department of Crop Science, College of Agriculture and Life & Environment Sciences, Chungbuk National University, Cheongju 28644, Republic of Korea; ygcho@chungbuk.ac.kr; 4 Institute of Genetic Engineering, Hankyong National University, Anseong 17579, Republic of Korea

**Keywords:** *OsGAPDH*, gene editing, CRISPR/Cas9, cytosolic, expression profile, GAPDH activity, free amino acid

## Abstract

Glyceraldehyde-3-phosphate dehydrogenase (GAPDH) is a major glycolytic enzyme that plays an important role in several cellular processes, including plant hormone signaling, plant development, and transcriptional regulation. In this study, we divided it into four groups through structural analysis of eight *GAPDH* genes identified in the rice genome. Among them, the expression level of five genes of cytosolic *GAPDH* was shown to be different for each organ. The mutation induction of the *GAPDHC7* gene by the CRISPR/Cas9 system revealed that the 7 bp and 2 bp deletion, early end codon, was used in protein production. In addition, the selected mutants showed lower plant heights compared to the wild-type plants. To investigate the effect on carbohydrate metabolism, the expression of the genes of starch-branched enzyme I (*SbeI*), sucrose synthase (SS), and 3-phosphoglycer phosphokinase (PGK) increased the expression of the *SBeI* gene threefold in the knockout lines compared to the wild-type (WT) plant, while the expression of the *SS* and *PGK* genes decreased significantly. And the starch and soluble sugar content of the knockout lines increased by more than 60% compared to the WT plant. Also, the free amino acid content was significantly increased in the Gln and Asn contents of the knockout lines compared to the WT plants, while the contents of Gly and Ser were decreased. Our results suggest that *OsGAPDHC7* has a great influence on energy metabolism, such as pre-harvested sprouting and amino acid content.

## 1. Introduction

Rice (*Oryza sativa* L.) is favored by more than half of the world’s population and is a very important food crop, especially in Asia and Africa. In recent years, crop yields have been rapidly decreasing in many countries around the world due to climate change and drought stress [1,2,3]. Glyceraldehyde-3-phosphate dehydrogenase (GAPDH), a key enzyme in the glycolytic pathway in plants, reversibly converts the glyceraldehyde-3-phosphate to 1,3-bisphosphoglycerate by coupling with the reduction in NAD1 to NADH [4]. *GAPDH* was once considered a simple “housekeeping gene” [5]. Therefore, it is often used as a reference gene to check the level of gene expression in plants [6]. In recent years, many researchers have reported on the important roles of *GAPDH* in intracellular abscisic acid (ABA) signaling, DNA repair, embryo development, energy production, sugar and amino acid balance, transcriptional regulation, and viable pollen development [7,8,9]. So far, a series of *GADPH* genes have been cloned in plants, and their characteristics have been reported [10,11,12]. The *GAPDH* gene was divided into isoforms depending on whether it is in the cytoplasm (Cy) and plastid (P) of the cell [13,14]. Of the many *GAPDH* genes in Arabidopsis, only the *GAPC1* and *GAPC2* genes are in the cytoplasm, while the rest of the genes are in the plastid [15,16]. Ref. [10] reported that oil content in seeds increased when the *GAPDH* gene was dropped into the cytoplasm. When the *GAPC1* gene was knocked down by the RNAi method in soybeans, the activity of nodule nitrogenase activity was greatly reduced [17]. Another study reported that plastidic GAPCP greatly influences the gene expression of starch biosynthetic metabolism [18]. Thus, the results reported so far suggest that the loss of function of the plastidic GAPCps gene will have a significant impact on plant growth and development. In this study, knockout mutants of *OsGAPDHC7* gene encoding plastidic glyceraldehyde-3-phosphate dehydrogenase were generated using the CRISPR/Cas9 system. Not only did these mutants affect plant growth and development, but they also showed changes in the content of amino acids and sugars compared to WT plants.

## 2. Results

### 2.1. Confirmation and Expression of OsGAPDHs in the Rice Genome

**Figure 1:**
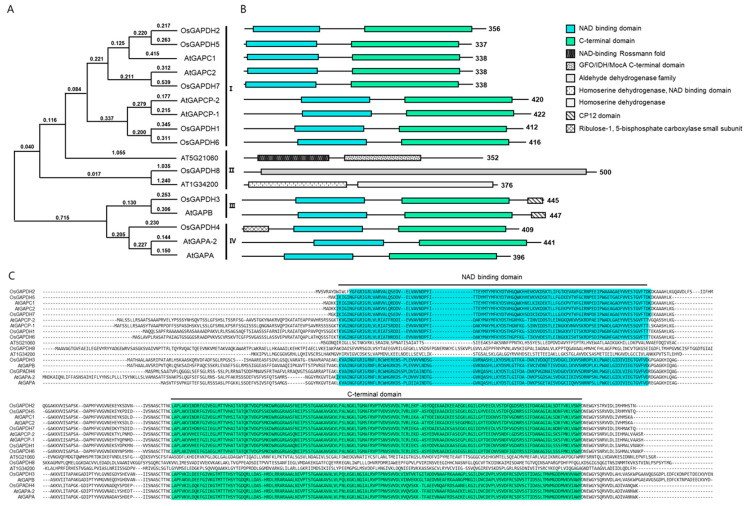
The phylogenetic tree and sequence alignment of GAPDH proteins. (**A**) Neighbor-joining phylogenetic tree of the GAPDH family. The phylogenetic analysis was represented using the MEGA 5.1 software. The four groups (I, II, II, IV) are as follows: I, cytosolic *GAPDH*; II, non-phosphorylated *GAPDH*; II, chloroplastic *GAPDHB* subunit; IV, chloroplastic *GAPDHA* subunit. (**B**) The predicted Gp_dh_N domain is blue, and the Gp_dh_C domain is green. (**C**) Multiple sequence alignment of the domain in rice and *Arabidopsis*.

**Figure 2:**
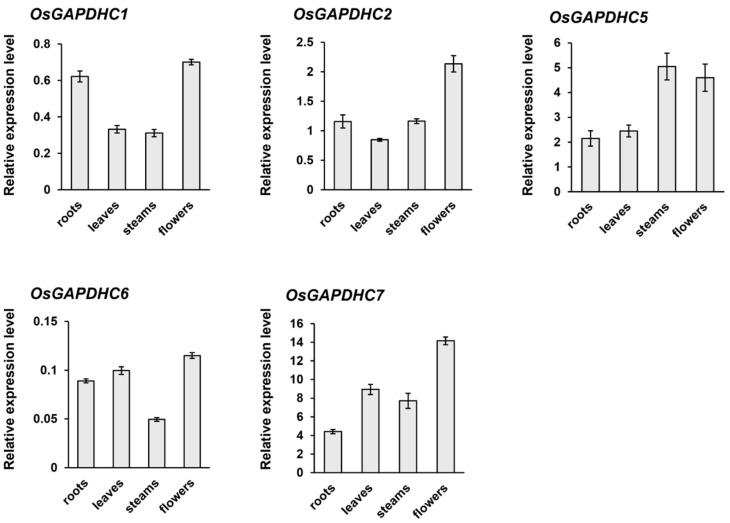
The expression patterns of *OsGAPDHC* genes. qPCR analysis expression of *OsGAPDHC* genes in four tissues of rice including roots, leaf, stem, and flower. Error bars represent standard deviations calculated in three replicates.

A GRAMENE website and database search revealed eight gene families encoding GAPDH in the rice genome. Phylogenetic analysis of these *GAPDH* genes showed that they could be classified into four major functional groups: chloroplastic *GAPDHA* subunit (GAPDHA), chloroplastic *GAPDHB* subunit (GAPDHB), cytosolic *GAPDH* (GAPDHC), and non-phosphorylated *GAPDH* (NP-GAPDH) (Figure 1A). Among them, there are five genes belonging to the *GAPDHC1* (LOC_Os02g07490), *OsGAPDHC2* (LOC_Os02g38920), *OsGAPDHC5* (LOC_Os04g40950), *OsGAPDHC6* (LOC Os06g45590), and *OsGAPDHC7* (LOC_Os08g03290). To determine the expression level of these five genes, total RNA was extracted from leaves, stems, roots, and flowers. qRT-PCR analysis showed that the expression level of genes in each institution was different (Figure 2). For the *OsGAPDHC7* gene, the expression level was in the order of flower > leaf > stem > root.

### 2.2. Generation of OsGAPDHC7 Knockout Homozygous Mutants

**Figure 3:**
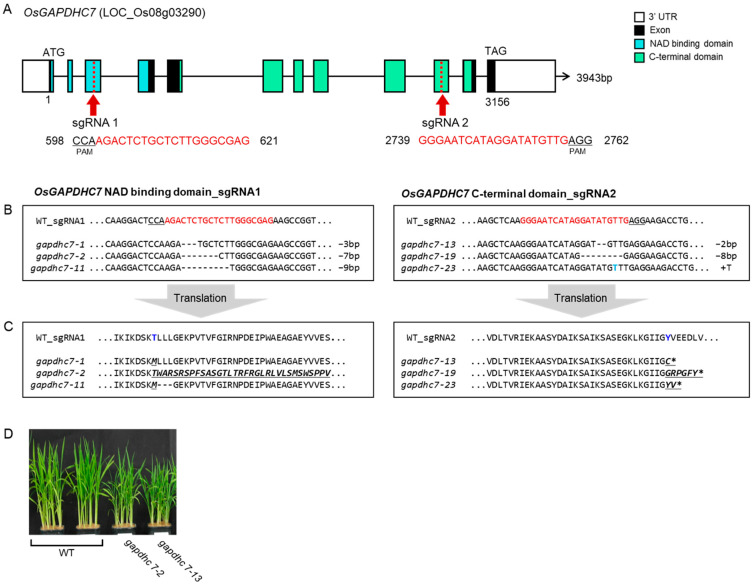
The *OsGAPDHC7* gene editing and sanger sequencing analysis. (**A**) Schematic diagram of the *OsGAPDHC7* gene with sgRNA designed in the NAD+ binding domain region and C-terminal domain region. (**B**) Sanger sequencing analysis results in the gene-edited plants. The underline indicates the PAM region. Deletion bases are indicated by “−” and their sizes, and insertion bases are indicated by “+” and their bases. Insertion bases are indicated in blue letters. Red arrows and letters indicate the positions and sequences of sgRNA. (**C**) Amino acid translation of edited regions. (**D**) The phenotype of *gapdhc7-2* and *gapdhc7-13* mutant lines and WT plants.

To investigate the in vivo function and role of *GAPDHC7* in rice, six transgenic plants were obtained by editing the third and tenth exons of *OsGAPDHC7* in Dongjin as a target using the CRISPR/Cas9 system (Supplementary Table S1). Two mutants were identified and harvested by target site sequencing (Figure 3A). Frame shift mutations with defects of 7 bp (CTCTGCT) and 2 bp (AT) in the *gapdhc7* homozygous mutant were identified by Sanger sequencing (Figure 3B). The predicted amino acid sequences of these mutants were examined, and as a result of the 7 bp and 2 bp deletions in the *GAPDHC7* gene, early end codon was caused in protein production (Figure 3C, Supplementary Figure S1). The phenotypes of the *gapdhc7-2* and *gapdhc7-13* lines showed significant differences in plant height compared to the WT plants (Figure 3C).

### 2.3. GAPDH Activity in the gapdhc7-2 and gapdhc7-13 Lines

**Figure 4:**
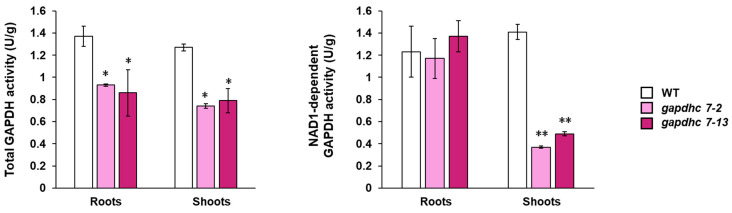
Change in GAPDH activity in the seedling of the *gapdhc7-2* and *gapdhc7-13* mutant lines and WT plants. Error bars represent standard deviations calculated in three replicates. Statistical significance was analyzed with Student’s *t*-test (** *p* < 0.05, * *p* < 0.1).

Despite the differences in the phenotypes of the *gapdhc7-2* and *gapdhc7-13* lines, the total GAPDH activity of the seedlings was not significantly different compared to that of the WT plants (Figure 4, Supplementary Table S2). However, in the plastid-enriched fractions of the *gapdhc7-2* and *gapdhc7-13* lines, the NAD1-dependent GAPDH activity was reduced by approximately 30% when compared to the WT plants (Figure 4, Supplementary Table S2).

### 2.4. The gapdhc7-2 and gapdhc7-13 Lines Affect Energy Metabolism

**Figure 5:**
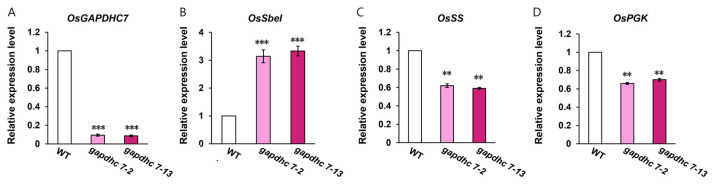
Changes in gene expression in the *gapdhc7-2* and *gapdhc7-13* mutant lines and WT plants. (**A**) *OsGAPDHC7* gene expression level assay. (**B**) Starch branching enzyme I (*OsSb*e*I*) expression level assay. (**C**) Sucrose synthase (*OsSS*) expression level assay. (**D**) 3-Phosphoglyceric phosphokinase (*PGK*) expression level assay. The immature samples were stored at −80 °C for 60 days and used in the experiments. Error bars represent standard deviations calculated in three replicates. Statistical significance was analyzed with Student’s *t*-test (*** *p* < 0.01, ** *p* < 0.05).

**Figure 6:**
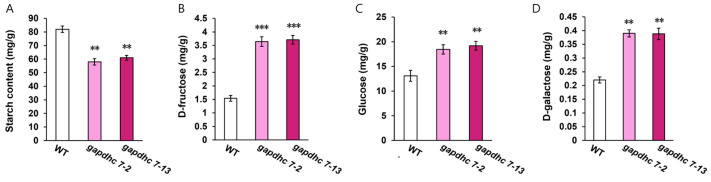
Changes in carbohydrate metabolism in *gapdhc7-2* and *gapdhc7-13* mutant lines and WT plants. (**A**) Starch contents analysis. (**B**) Fructose contents analysis. (**C**) Glucose contents analysis. (**D**) Galactose contents analysis. Seeds were stored at room temperature and used in experiments. Error bars represent standard deviations calculated in three replicates. Statistical significance was analyzed with Student’s *t*-test (*** *p* < 0.01, ** *p* < 0.05).

Destruction of the *GAPDHC* gene in the rice genome may lead to damage to lipids, carbohydrate, and amino acid metabolism. To investigate the effect of *GAPDHC7* on carbohydrate metabolism, we first performed qRT-PCR analysis of the gene expression of starch-branched enzyme I (*SbeI*), sucrose synthase (*SS*), and 3-phosphoglycer phosphokinase (*PGK*). As a result, the expression of the *SBeI* gene in the *gapdhc7-2* and *gapdhc7-13* lines increased threefold compared to that in the WT plants, whereas the expression of the *SS* and *PGK* genes was significantly decreased (Figure 5). In addition, the starch and soluble sugar contents of the *gapdhc7-2* and *gapdhc7-13* lines increased by about 60% or more compared to the WT plants (Figure 6).

### 2.5. Change in Free Amino Acid in the gapdhc7-2 and gapdhc7-13 Lines

Free amino acid content was analyzed using the roots and seedlings of the *gapdhc7-2* and *gapdhc7-13* lines. As a result, the contents of Gln, Asn, Gly, and Ser in the *gapdhc7-2* and *gapdhc7-13* lines were changed compared to the WT plants. Among them, the contents of Gln and Asn in the *gapdhc7-*2 and *gapdhc7-13* lines were significantly increased compared to the WT plants, while the contents of Gly and Ser were decreased (Table 1). In the roots of the *gapdhc7-2* and *gapdhc7-13* lines, the contents of Gln, Asn, Gly, and Ser were significantly increased compared to the WT plants.

## 3. Discussion

Targeted genome editing using the CRISPR/Cas9 system has been applied to many plants for gene function research and the improvement of agricultural characteristics. Gene editing using the CRISPR/Cas9 system is accurate, simple, and very easy to use [19], and can dispel consumer concerns about genetically modified organisms [20]. Therefore, in this study, the physiological role of the *OsGAPDHC7* gene associated with plastidic glyceraldehyde-3-phosphate dehydrogenase was studied using genome-edited lines. GAPDHC is involved in plastid glycolysis pathways and plays a very important role in the generation of glycolysis energy in non-green plastids and chloroplasts [21]. We have shown that the phylogenetic analysis of *GAPDH* genes in rice genomes can be classified into four functional groups: chloroplastic GAPDHA (GAPDHA), chloroplastic GAPDH B subunit (GAPDHB), cytosolic GAPDH (GAPDHC), and non-phosphorylated GAPDH (NP-GAPDH) (Figure 1A). Among them, the *OsGAPDHC7* gene encoding the plastidial isoform can correspond to isoforms that were biochemically described in other plant species [22]. Therefore, in this study, the knockout line obtained by the CRISPR/Cas9 system of the *OsGAPDHC7* gene significantly decreased the growth of seedlings and roots compared to the WT plants (Figure 2). So far, dwarfism and infertility have occurred in *GAPDHC* mutants due to changes in glycolysis and crab cycle intermediates, and decreased GAPDH activity [22]. In addition, the inhibition of the expression of the potato *GAPDHC* gene by the antisense method had little effect on plant morphology and metabolic regulation [23]. Reference [24] reported that the major glycolytic pool between cytoplasm and plastids remains in equilibrium. Therefore, it is known that inhibiting the expression of the *GAPDHC* gene in potatoes did not cause serious phenotypic changes [24]. In the double mutants of *GAPDHC* genes in *Arabidopsis*, the phenotype was dwarfed and infertile, and the process product was not in equilibrium between cytoplasm and plastid [18]. Similarly, in our results, despite the differences in the phenotypes of the *gapdhc7-2* and *gapdhc7-13* lines, total GAPDH activity in the seedlings did not show significant differences compared to WT plants (Table 1). These results suggest that glycolysis products are not in equilibrium between the cytoplasm and plastids. Carbohydrates such as starch, sucrose, and glucose are necessary for plant growth and development, and high levels of sucrose and ATP affect the promotion of seedling growth [25]. Destruction of the *GAPDHC* gene in the rice genome can lead to damage to lipids, carbohydrate, and amino acid metabolism. In the present study, the expression of starch-branched enzyme I (*SbeI*) in the *gapdhc7-2* and *gapdhc7-13* lines was increased threefold compared to the control plant, and the expression of sucrose synthase (*SS*) and 3-phosphoglycer phosphokinase (*PGK*) was significantly decreased compared to the WT plants (Figure 4). Collectively, the *gapdhc7-2* and *gapdhc7-13* lines had higher starch contents than the WT, and glucose, galactose, and sucrose contents were lower than those of the WT. Therefore, this suggests that disruption of the *GAPDHC1* gene resulted in large changes in the energy metabolism involved in growth and development. Starch is only synthesized and degraded in plastids, but carbon obtained by starch degradation is added as hexose to the glycolysis of the cytoplasm [26]. Therefore, the blockade of the plastid glycolysis pathway of the *gapcpc1* mutation may be short-circuited through the cytoplasmic pathway, in which highly active GAPDHC may metabolize trisaccharide phosphate. Thereafter, the metabolite may re-enter the plastid pathway with pyruvate, 3-PGA, or PEP. Starch and glycogen are the main storage carbohydrates of plants and bacteria, respectively [27]. The metabolism of these reserve polysaccharides is well regulated within cells and is known to be related to amino acid metabolism in response to physiological needs [28]. In *Escherichia coli*, amino acid deficiency induces a “strict response,” a multiple physiological change that downregulates nucleic acid and protein synthesis and upregulates the expression of genes involved in glycogen biosynthesis. In this paper, changes in GAPDH activity, amino acid, and starch biosynthesis in the *gapdhc7-2* and *gapdhc7-13* lines generated by gene editing played an important role in regulating energy metabolism, especially carbon metabolism. Therefore, this suggests that the *GAPDHC7* gene is an important factor for starch accumulation as well as amino acid homeostasis.

## 4. Materials and Methods

### 4.1. Plant Materials

Dongjin, a rice variety, was used as a transformation receptor and control. Seedlings were grown by transplanting at intervals of 30×15 cm in glass greenhouses. The cultivation management was carried out according to the rice cultivation standards adapted to the experimental area of Hankyoung National University, by applying N-P_2_O_5_-K_2_O fertilizer at a ratio of 90—45–47 kg/ha.

### 4.2. Phylogenetic Analysis

The phylogenetic analysis was generated according to the estimate of the evolutionary divergence between *GAPDH* genes in rice and *Arabidopsis thaliana*. All information was searched using database such as RAP-DB, NCBI “https://blast.ncbi.nlm.nih.gov/Blast.cgi”, and Gramene. In addition, the evolutionary history was constructed by the neighbor-joining method and MEGA 7 software [29].

### 4.3. Gene Editing

Based on the *GAPDHC7* sequence provided by the NCBI database (https://blast.ncbi.nlm.nih.gov), two target sites were designed in the first exon region of *GAPDHC7* adjacent to a protospacer-adjacent motif (PAM), using the CRISPR RGEN tool (http://www.rgenome.net/ accessed on 2 September 2022) developed by the Hanyang University [30]. For CRISPR target sequences regarding cloning, annealed pairs of oligonucleotides, synthesized by Bioneer Co., Ltd. (Dajeon, Republic of Korea), were cloned to pBOsC by an *Aar* I cut. The constructs thus obtained were transformed into a rice embryogenic callus using the *Agrobacterium tumefaciens* strain EHA105, as previously described [31]. The transformed callus was selected using 6 mg/L of phosphinothricin and confirmed by PCR analysis, as previously reported [32]. To verify the target site mutation, PCR reactions were subjected to MiniSeq paired-end read sequencing (Illumina, San Diego, CA, USA) and analyzed using the Cas-Analyzer (https://www.rgenome.net/cas-analyzer/#!, accessed on 4 September 2022) [32,33]. All transgenic callus lines performed several subcultures and were maintained in 2N6 medium (Supplementary Figure S2), as described previously [34]. The regenerated plants were moved to pots in the greenhouse. Genomic DNA extraction and deep sequencing analysis were performed as previously reported by Jung et al. [32]. The NGS data were analyzed using Cas-Analyzer (https://www.rgenome.net/cas-analyzer, accessed on 5 April 2023) [32,33,34].

### 4.4. Detection of Mutation Type

The standard PCR conditions were as follows: 94 °C for 3 min; 94 °C for 40 s; 56 °C for 40 s; 72 °C for 50 s for 35 cycles; and 72 °C for 10 min. The PCR products were directly sequenced by NGS technology using internal sequencing primers (Supplementary Table S3) to identify mutations.

### 4.5. qRT-PCR Analysis

Reverse transcription was conducted using reverse transcription kits provided by Promega (Seoul, Republic of Korea) Co., Ltd., and quantitative RT-PCR was conducted using Promega^®^ GoTaq^®^ qPCR Probe kit (Seoul, Republic of Korea). The Actin gene was used as the internal control for expression analysis, conducted using a BIO RAD real-time fluorescence quantitative PCR instrument. The reaction was performed with biological triplicates as described previously [32,35]. Relative gene expression was calculated using the following formula: Relative Expression = 2^−ΔΔCT^. Primer sequences for RT-PCR analysis are listed in Supplementary Table S3.

### 4.6. GAPDH Activity and Enzyme Analysis

GAPDH activity was measured by dividing into whole and plastid extracts according to a method previously reported by [18]. Protein content was quantified using a Bio-Rad protein analysis kit. GAPDH activity in the extract was analyzed according to a method reported by [36].

### 4.7. Analysis of Carbohydrate and Amino Acid

Starch, glucose, galactose, and sucrose contents were analyzed with the ENZYTECTM Generic starch kit (R-Biopharm, Darmstadt, Germany). Amino acid content was analyzed by the Korea Basic Science Institute (KBSI), Daejeon, Republic of Korea. The amino acid composition analysis comprised an HPLC system (separation module), a UV/visible detector, a fluorescence detector, and chromatography separations. Detailed protocols for HPLC-MS/MS metrology, chromatography, and MS parameters were applied according to previously reported methods [37].

## Figures and Tables

**Table 1:** Analysis of free amino acid content of *gapdhc7-2* and *gapdhc7-13* mutant lines and WT plants.

Amino Acid	WT	*grpdhc 7-2*	*grpdhc 7-13*
Asp	157.2 ± 6.8	149.3 ± 4.4	162.4 ± 6.7
Asn	20.4 ± 2.2	67.4 ± 3.2	72.7 ± 2.9
Phe	12.4 ± 3.2	14.2 ± 1.8	13.2 ± 3.2
Val	85.4 ± 6.7	80.8 ± 6.1	86.3 ± 7.2
Lys	28.2 ± 1.9	30.2 ± 3.0	27.4 ± 3.2
Ala	66.7 ± 3.2	71.2 ± 3.1	68.5 ± 4.2
Tyr	20.4 ± 1.8	19.8 ± 2.2	22.4 ± 2.4
His	40.4 ± 0.9	38.9 ± 1.9	41.3 ± 2.1
Met	90.4 ± 4.15	89.7 ± 1.9	92.3 ± 7.2
Glu	201.0 ± 52.4	198.7 ± 22.1	203.0 ± 38.3
Pro	53.9 ± 3.7	62.3 ± 7.8	50.3 ± 11.2
Gly	20.4 ± 2.7	9.7 ± 3.1	7.8 ± 1.8
Ile	74.2 ± 8.5	72.4 ± 3.1	75.4 ± 2.8
Leu	85.4 ± 10.1	81.3 ± 2.9	86.4 ± 7.2
Arg	24.9 ± 1.8	24.7 ± 2.1	19.3 ± 7.0
Thr	35.9 ± 1.2	32.7 ± 2.2	36.7 ± 2.7
Gln	71.7 ± 12.4	137.5 ± 2.4	142.8 ± 12.1
Ser	107.3 ± 10.3	67.7 ± 7.8	52.8 ± 7.9

Asp: Aspartic acid; Asn, Asparagine; Phe, Phenylalanine; Val, Valine; Lys, Lysine; Ala, Alanine; Tyr, Tyrosine; His, Histidine; Met, Methionine; Glu, Glutamic acid; Pro, Proline; Gly, Glycine; Ile, Isoleucine; Leu, Leucine; Arg, Arginine; Thr, Threonine; Gln, Glutamine; Ser, Serine.

## Data Availability

The original contributions presented in the study are included in the article/Supplementary Materials, further inquiries can be directed to the corresponding authors.

## Supplementary Materials

The following supporting information can be downloaded at https://www.mdpi.com/article/10.3390/ijms252212470/s1.
