# Supplementary material for: Knockout of OsGAPDHC7 Gene Encoding Cytosolic Glyceraldehyde-3-Phosphate Dehydrogenase Affects Energy Metabolism in Rice Seeds
Source: Int J Mol Sci. 2024 Nov 20;25(22):12470. doi: 10.3390/ijms252212470 (PMC11594994; doi:10.3390/ijms252212470)
Supplement: Supplementary file 1 [file ijms-25-12470-s001.zip › ijms-3303963-supplementary.pdf]

Supplementary Data

Knockout of *OsGAPDHC7* Gene Encoding Plastidial Glyceraldehyde-3-Phosphate Dehydrogenase Affects Energy Metabolism in Rice Seeds

Jin Young Kim [1], Ye Ji Lee [1], Hyo Ju Lee [1], Ji Yun Ko [3], Hae Mi Lee [1], Jin Shil Park [1], Yong-Gu Cho[3]Y, Yu Jin Jung[1,4,] and Kwon Kyoo Kang [1,4,*]

Contents

Table S1. Mutation frequencies at on-target and potential off-target sites among selected sgRNA target of the *OsGAPDHC7* genes in rice genome using RGEN tools (http://www.rgenome.net/cas-designer/).

Table S2. GAPDH enzyme activity assay in roots and shoots of WT and *gapdhc7* mutants.

Table S3. The primers list used in this study.

Figure S1. Amino acid sequences of WT and *gapdhc7* mutants. "-" indicates deletion, "*" indicates stop codon. Blue letters indicate target positions, and bold underlined letters indicate amino acid sequences changed by frame shift through gene editing.

Figure S2. Breeding process of *gapdhc7* transgenic plants. (A) Schematic diagram of pBOsC Vector for monocot transformation. sgRNA is integrated into the vector via the *Aar* I restriction enzyme site. (B) Production of transgenic plants through tissue culture. Shoots and roots are generated from rice calli grown in 2N6 medium, which develop into regenerated plants. (C) Selection of transgenic plants through PCR analysis.

T-DNA insertion is confirmed in regenerated plants by amplifying the *bar* gene/nos terminator region in pBOsC vector.

Supplementary Table S1. Mutation frequencies at on-target and potential off-target sites among selected sgRNA target of the *OsGAPDHC7* genes in rice genome using RGEN tools (http://www.rgenome.net/cas-designer/).

	RGEN Target (5' to 3')	Direction	GC Contents(%, w/o PAM)	Out-of-frame Score	Mismatches 0	1	2	3
**sgRNA1**	GCGTATTGCAACCAGTAGAGAGG	-	50	50	1	0	0	0
**sgRNA2**	CTGTTGTCGACTTGACCTGCCGG	+	55	67.6	1	0	0	0

Supplementary Table S2. GAPDH enzyme activity assay in roots and shoots of WT and *gapdhc7* mutants.

Line	Total GAPDH activity (U/g)		NAD1-dependent GAPDH activity (U/g)	
	roots	shoots	roots	shoots
WT	1.37 ± 0.09	1.27 ± 0.03	1.23 ± 0.23	1.41 ± 0.07
gapdhc 7-2	0.93 ± 0.01	0.74 ± 0.02	1.17 ± 0.18	0.37 ± 0.01
gapdhc 7-13	0.86 ± 0.21	0.79 ± 0.11	1.37 ± 0.14	0.49 ± 0.02

Supplementary Table S3. The primers list used in this study.

Primer name	Sequence (primer direction 5′-3′)	purpose
J67 pBOsC sgSEQ - FW	CAGCTTGGCTCTAGTCGACC	Vector construction
K20 RGEN scaaffold regoin RV	CGGTGCCACTTTTTCAAGTT	
OsGAPDHC7 NAD binding domain_sg1 up	ggcagCTCGCCCAAGAGCAGAGTCT	
OsGAPDHC7 NAD binding domain_sg1 down	aaacAGACTCTGCTCTTGGGCGAGc	
OsGAPDHC7 C-terminal domain_sg2 up	ggcagGGGAATCATAGGATATGTTG	
OsGAPDHC7 C-terminal domain_sg2 down	aaacCAACATATCCTATGATTCCCc	
T-DNA confirm-Nos ter Fw	TTGCGCGCTATATTTTGTTTT	T-DNA confirm
T-DNA confirm-Bar R Rv	CGTCAACCACTACATCGAGA	
OsGAPDHC7 NAD_sg1 1st F1	TGCTCGCTCAAAGAGATGTT	deep-sequencing
OsGAPDHC7 NAD_sg1 1st R1	TGCCAATTTGTTGGAATGAA	
OsGAPDHC7 NAD_sg1 2nd F1	ACACTCTTTCCCTACACGACGCTCTTCCGATCTGGCTTTGATTTTCGTGTGGT	
OsGAPDHC7 NAD_sg1 2nd R1	GTGACTGGAGTTCAGACGTGTGCTCTTCCGATCTCACGCTCGCTACCATAAACA	
OsGAPDHC7 C-terminal_sg2 1st F1	GACGAATTGCTTGCTGTTCA	
OsGAPDHC7 C-terminal_sg2 1st R1	TGCTCGACCTGAGTCAAAGA	
OsGAPDHC7 C-terminal_sg2 2nd F1	ACACTCTTTCCCTACACGACGCTCTTCCGATCTGCGCAGAACTGCACAAGTTA	
OsGAPDHC7 C-terminal_sg2 2nd R1	GTGACTGGAGTTCAGACGTGTGCTCTTCCGATCTCGTAACCACTTAGTTTGGAGCA	
OsACTIN FW	CAACACCCCTGCTATGTACG	qRT-PCR analysis
OsACTIN RV	ATCACCAGAGTCCAACACAA	
OsGAPDHC1 qRT-PCR Fw	TTGACCTGCCGGATTGAAAA	
OsGAPDHC1 qRT-PCR Rv	TGCATCACCAACGAGTCATT	
OsGAPDHC2 qRT-PCR Fw	AGCTGACTGGAATGGCTTTC	
OsGAPDHC2 qRT-PCR Rv	CCCTCAGCCTCCTACTTGAT	

OsGAPDHC5 qRT-PCR Fw	CAGAAGACCGTTGATGGACC
OsGAPDHC5 qRT-PCR Rv	CGGAAAGCCATACCAGTCAA
OsGAPDHC6 qRT-PCR Fw	GTCGACGGTCCTTCAATGAA
OsGAPDHC6 qRT-PCR Rv	CCATACCAGTGAGTTTCCCG
OsGAPDHC7 qRT-PCR Fw	CCCAAAAGACCGTTGATGGA
OsGAPDHC7 qRT-PCR Rv	ATTCCCGTAAGCTTGCCATT
OsSBEI qRT-PCR Fw	GGCATTGCACTCCAAAAGAT
OsSBEI qRT-PCR Rv	GCTCCAGTTGTTGCCTTCTC
OsSS qRT-PCR Fw	TCCGAGAGGTTCAGGTCATC
OsSS qRT-PCR Rv	ATGAGCTCCTCGGCGTAGTA
OsPGK qRT-PCR Fw	TGGGGTGATTGAGTCTCTGT
OsPGK qRT-PCR Rv	AAGAGAGAAACACCCTTCGC

A

```
WT           1   MGKIKIGINGFGRIGRLVARVALQSEDVELVAVNDPFITTDYMTYMFKYDTVHGQWKHSDIKIKDSKTLLLGEKPVTVFGIRNPDEIPWAEAGAEYVVES  100
gapdhc 7-1   1   MGKIKIGINGFGRIGRLVARVALQSEDVELVAVNDPFITTDYMTYMFKYDTVHGQWKHSDIKIKDSKMLLLGEKPVTVFGIRNPDEIPWAEAGAEYVVES  100
gapdhc 7-2   1   MGKIKIGINGFGRIGRLVARVALQSEDVELVAVNDPFITTDYMTYMFKYDTVHGQWKHSDIKIKDSKTWARSRSPFSASGTLTRFRGLRLVLSMSWSPPV  100
gapdhc 7-11  1   MGKIKIGINGFGRIGRLVARVALQSEDVELVAVNDPFITTDYMTYMFKYDTVHGQWKHSDIKIKDSKM---GEKPVTVFGIRNPDEIPWAEAGAEYVVES  97

WT           101 TGVFTDKEKAAAHLKGGAKKVVISAPSKDAPMFVCGVNEDKYTSDIDIVSNASCTTNCLAPLAKVIHDNFGIIEGLMTTVHAITATQKTVDGPSSKDWRG  200
gapdhc 7-1   101 TGVFTDKEKAAAHLKGGAKKVVISAPSKDAPMFVCGVNEDKYTSDIDIVSNASCTTNCLAPLAKVIHDNFGIIEGLMTTVHAITATQKTVDGPSSKDWRG  200
gapdhc 7-2   101 SSLTRRRLLLT*                                                                                          111
gapdhc 7-11  98  TGVFTDKEKAAAHLKGGAKKVVISAPSKDAPMFVCGVNEDKYTSDIDIVSNASCTTNCLAPLAKVIHDNFGIIEGLMTTVHAITATQKTVDGPSSKDWRG  197

WT           201 GRAASFNIIPSSTGAAKAVGKVLPDLNGKLTGMSFRVPTVDVSVVDLTVRIEKAASYDAIKSAIKSASEGKLKGIIGYVEEDLVSTDFVGDSRSSIFDAK  300
gapdhc 7-1   201 GRAASFNIIPSSTGAAKAVGKVLPDLNGKLTGMSFRVPTVDVSVVDLTVRIEKAASYDAIKSAIKSASEGKLKGIIGYVEEDLVSTDFVGDSRSSIFDAK  300

gapdhc 7-11  198 GRAASFNIIPSSTGAAKAVGKVLPDLNGKLTGMSFRVPTVDVSVVDLTVRIEKAASYDAIKSAIKSASEGKLKGIIGYVEEDLVSTDFVGDSRSSIFDAK  297

WT           301 AGIALNDNFVKLVAWYDNEWGYSNRVIDLIRHMAKTQ*                                                                337
gapdhc 7-1   301 AGIALNDNFVKLVAWYDNEWGYSNRVIDLIRHMAKTQ*                                                                337

gapdhc 7-11  298 AGIALNDNFVKLVAWYDNEWGYSNRVIDLIRHMAKTQ*                                                                335
```

B

```
WT           1   MGKIKIGINGFGRIGRLVARVALQSEDVELVAVNDPFITTDYMTYMFKYDTVHGQWKHSDIKIKDSKTLLLGEKPVTVFGIRNPDEIPWAEAGAEYVVES  100
gapdhc 7-13  1   MGKIKIGINGFGRIGRLVARVALQSEDVELVAVNDPFITTDYMTYMFKYDTVHGQWKHSDIKIKDSKTLLLGEKPVTVFGIRNPDEIPWAEAGAEYVVES  100
gapdhc 7-19  1   MGKIKIGINGFGRIGRLVARVALQSEDVELVAVNDPFITTDYMTYMFKYDTVHGQWKHSDIKIKDSKTLLLGEKPVTVFGIRNPDEIPWAEAGAEYVVES  100
gapdhc 7-23  1   MGKIKIGINGFGRIGRLVARVALQSEDVELVAVNDPFITTDYMTYMFKYDTVHGQWKHSDIKIKDSKTLLLGEKPVTVFGIRNPDEIPWAEAGAEYVVES  100

WT           101 TGVFTDKEKAAAHLKGGAKKVVISAPSKDAPMFVCGVNEDKYTSDIDIVSNASCTTNCLAPLAKVIHDNFGIIEGLMTTVHAITATQKTVDGPSSKDWRG  200
gapdhc 7-13  101 TGVFTDKEKAAAHLKGGAKKVVISAPSKDAPMFVCGVNEDKYTSDIDIVSNASCTTNCLAPLAKVIHDNFGIIEGLMTTVHAITATQKTVDGPSSKDWRG  200
gapdhc 7-19  101 TGVFTDKEKAAAHLKGGAKKVVISAPSKDAPMFVCGVNEDKYTSDIDIVSNASCTTNCLAPLAKVIHDNFGIIEGLMTTVHAITATQKTVDGPSSKDWRG  200
gapdhc 7-23  101 TGVFTDKEKAAAHLKGGAKKVVISAPSKDAPMFVCGVNEDKYTSDIDIVSNASCTTNCLAPLAKVIHDNFGIIEGLMTTVHAITATQKTVDGPSSKDWRG  200

WT           201 GRAASFNIIPSSTGAAKAVGKVLPDLNGKLTGMSFRVPTVDVSVVDLTVRIEKAASYDAIKSAIKSASEGKLKGIIGYVEEDLVSTDFVGDSRSSIFDAK  300
gapdhc 7-13  201 GRAASFNIIPSSTGAAKAVGKVLPDLNGKLTGMSFRVPTVDVSVVDLTVRIEKAASYDAIKSAIKSASEGKLKGIIGC*                       237
gapdhc 7-19  201 GRAASFNIIPSSTGAAKAVGKVLPDLNGKLTGMSFRVPTVDVSVVDLTVRIEKAASYDAIKSAIKSASEGKLKGIIGGRPGFY*                  242
gapdhc 7-23  201 GRAASFNIIPSSTGAAKAVGKVLPDLNGKLTGMSFRVPTVDVSVVDLTVRIEKAASYDAIKSAIKSASEGKLKGIIGYV*                      238

WT           301 AGIALNDNFVKLVAWYDNEWGYSNRVIDLIRHMAKTQ*                                                                337
gapdhc 7-13
gapdhc 7-19
gapdhc 7-23
```

Supplementary Figure S1. Amino acid sequences of WT and *gapdhc7* mutants. "-" indicates deletion, "*" indicates stop codon. Blue letters indicate target positions, and bold underlined letters indicate amino acid sequences changed by frame shift through gene editing.

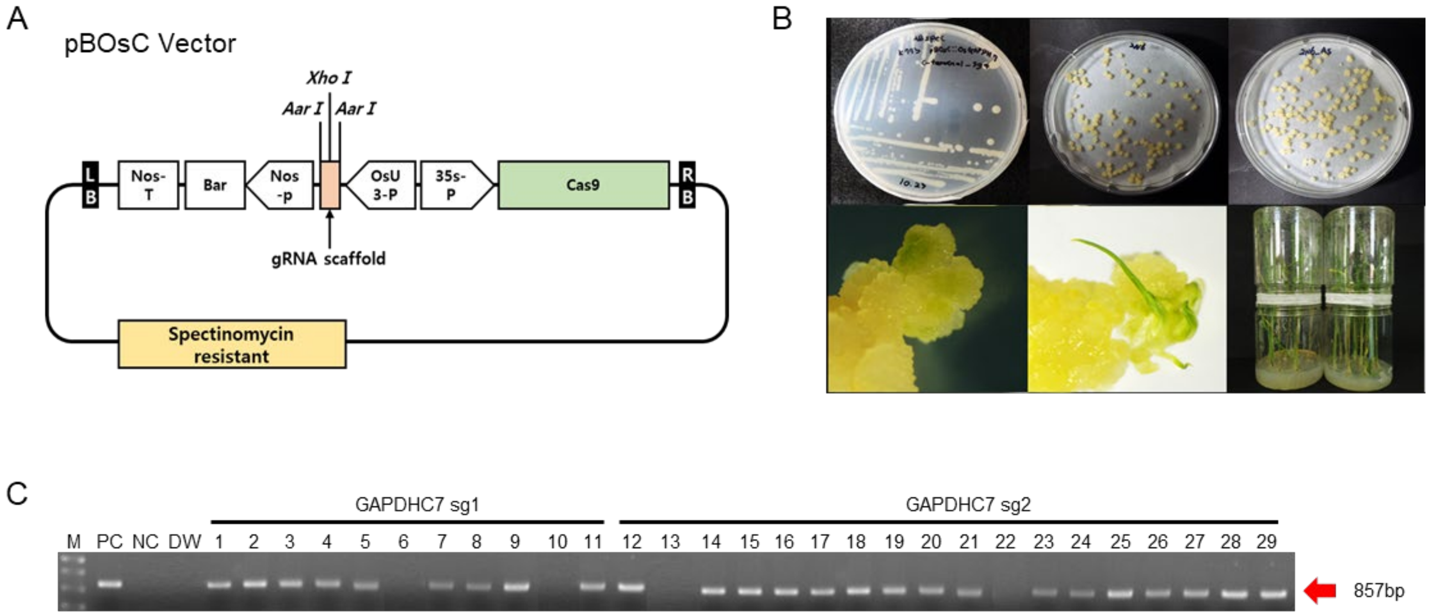

Supplementary Figure S2. Breeding process of *gapdhc7* transgenic plants. (A) Schematic diagram of pBOsC Vector for monocot transformation. sgRNA is integrated into the vector via the *Aar* I restriction enzyme site. (B) Production of transgenic plants through tissue culture. Shoots and roots are generated from rice calli grown in 2N6 medium, which develop into regenerated plants. (C) Selection of transgenic plants through PCR analysis. T-DNA insertion is confirmed in regenerated plants by amplifying the *bar* gene/nos terminator region in pBOsC vector.
